# Spinal Cord Segmentation by One Dimensional Normalized Template Matching: A Novel, Quantitative Technique to Analyze Advanced Magnetic Resonance Imaging Data

**DOI:** 10.1371/journal.pone.0139323

**Published:** 2015-10-07

**Authors:** Adam Cadotte, David W. Cadotte, Micha Livne, Julien Cohen-Adad, David Fleet, David Mikulis, Michael G. Fehlings

**Affiliations:** 1 Department of Surgery, Division of Neurosurgery, University of Toronto, Toronto, Ontario, Canada; 2 Toronto Western Hospital, University Health Network, Toronto, Ontario, Canada; 3 Department of Computer Science, University of Toronto, Toronto, Ontario, Canada; 4 Institute of Biomedical Engineering, Ecole Polytechnique de Montréal, Montreal, Quebec, Canada; 5 Functional Neuroimaging Unit, CRIUGM, Université de Montréal, Montreal, Quebec, Canada; 6 Department of Medical Imaging, Division of Neuroradiology, University of Toronto, Toronto, Ontario, Canada; 7 University of Toronto Spine Program, University of Toronto, Toronto, Ontario, Canada; Henry Jackson Foundation, UNITED STATES

## Abstract

Spinal cord segmentation is a developing area of research intended to aid the processing and interpretation of advanced magnetic resonance imaging (MRI). For example, high resolution three-dimensional volumes can be segmented to provide a measurement of spinal cord atrophy. Spinal cord segmentation is difficult due to the variety of MRI contrasts and the variation in human anatomy. In this study we propose a new method of spinal cord segmentation based on one-dimensional template matching and provide several metrics that can be used to compare with other segmentation methods. A set of ground-truth data from 10 subjects was manually-segmented by two different raters. These ground truth data formed the basis of the segmentation algorithm. A user was required to manually initialize the spinal cord center-line on new images, taking less than one minute. Template matching was used to segment the new cord and a refined center line was calculated based on multiple centroids within the segmentation. Arc distances down the spinal cord and cross-sectional areas were calculated. Inter-rater validation was performed by comparing two manual raters (n = 10). Semi-automatic validation was performed by comparing the two manual raters to the semi-automatic method (n = 10). Comparing the semi-automatic method to one of the raters yielded a Dice coefficient of 0.91 +/- 0.02 for ten subjects, a mean distance between spinal cord center lines of 0.32 +/- 0.08 mm, and a Hausdorff distance of 1.82 +/- 0.33 mm. The absolute variation in cross-sectional area was comparable for the semi-automatic method versus manual segmentation when compared to inter-rater manual segmentation. The results demonstrate that this novel segmentation method performs as well as a manual rater for most segmentation metrics. It offers a new approach to study spinal cord disease and to quantitatively track changes within the spinal cord in an individual case and across cohorts of subjects.

## Introduction

Imaging of the human central nervous system has made significant advances over the last decade, although acquisition and processing of spinal cord MRI data has lacked considerably relative to brain imaging. Two of the main reasons for this are challenges associated with data acquisition and difficulties associated with segmenting the spinal cord due to the natural variability in human spinal column anatomy. Different images present different segmentation challenges, including low image resolution, low cerebral spinal fluid (CSF) to spinal cord contrast, subjects with reduced CSF volume, subjects with above-average curvature of their spine, and artifacts due to patient motion from breathing and swallowing during image acquisition. Many segmentation methods have been developed over the last 20 years which attempt to resolve these difficulties. The existing methods vary in several ways, including fully automatic [1, 2], versus semi-automatic methods, the type of image contrast that can be handled (e.g., T1-weighted vs. T2-weighted), and the underlying mechanism of segmentation (e.g., active contour, atlas-based, deformable mesh, histogram-based, etc.). Summaries of the most widely used methods can be found in Chen et al., 2013 [3] and De Leener et al., 2014 [2]. The common goal across segmentation methods is to accurately define and distinguish the 3D boundaries of the spinal cord from the surrounding CSF, soft tissue, and vertebral bodies. Knowing this information can be useful in quantifying spinal cord atrophy [4, 5], surgical planning [6], locating functional areas of the spinal cord, such as nerve fiber tracts [7], and to locate precise anatomical regions of the spinal cord [8]. There is little debate regarding the potential utility of having a properly segmented spinal cord, but there is debate on the accuracy and efficiency of the various segmentation methods. No one method is able to perfectly accommodate every image consideration. It is likely that multiple segmentation modalities may be beneficial for many subjects due to the variation in spinal column anatomy and the difficulties faced with image acquisition.

One set-back faced in spinal cord segmentation methods is the lack of consensus regarding the measurement of segmentation accuracy. There are several metrics used to validate segmentation, including the Dice coefficient [9], the Hausdorff distance, and cross-sectional area (CSA). A combination of these metrics have been used to validate segmentations in numerous studies. [2, 3, 10–13] One less common validation tool, the mean correlation between manual and automated volume segmentations of the spinal cord, has been used, although the calculation was not explained in detail by the author. [6] Each of these techniques measures a different aspect of an automatic or semi-automatic segmentation method compared to a manual segmentation. Dice coefficient measures the overlap of two volumes, Hausdorff distance is the maximum distance between two volumetric surfaces, and CSA can be used to compare the 2D cross-sectional area in a particular region of a segmented volume. It is not clear how most of the prior studies compare on all of these metrics, as there is no standard to report all metrics. De Leener was the first to compare multiple metrics on a new segmentation method, compared to both manual segmentation and the Horsfield method. [2] To advance the field of spinal cord segmentation, further validation of both existing and new techniques is essential.

In this study, we report on the development of a novel method of spinal cord segmentation based on one-dimensional template matching. This method utilizes ground-truth data, validated by a human expert in spinal cord anatomy, to segment new images. It aims to be flexible regarding the curvature of the spinal cord, the amount of CSF present, the presence of motion and acquisition artifacts, and to require less than two minutes in processing time. The tools developed are intended for calculating arc distance along the spinal cord, cross-sectional area, and the center of the spinal cord for mapping purposes. To accomplish this we used a semi-automatic method which requires a small amount of user input to initialize the segmentation. A B-spline curve was used to represent the center-line of the spinal cord from which arc distance measurements can be made. From this center-line, comparisons of radial gradient magnitude values are made with corresponding ground-truth data, using one-dimensional template matching, to determine where the edge of the CSF / spinal cord lies on new images. Using the segmented image, a more refined spinal cord center-line can be calculated, then volume, cross-sectional area and distance measurement can be made within any volume of the spinal cord. Only one prior study utilized the B-spline approach, but used an active surface method rather than 1-D template matching. [4] We chose to use one-dimensional matching rather than two- or three-dimensional matching as it is likely to reduce the complexity of the calculations and processing time. This work has important practical clinical and research implications as it will enable an accurate mapping of the human spinal cord based on MR images.

## Materials and Methods

### Ethics Statement

MRI data was collected from ten healthy volunteers who were recruited through posted signs at the Toronto Western Hospital. Written, informed consent was obtained in all cases and approval to conduct this work was granted by our institutional ethics review board, the University Health Network Research Ethics Board (UHN-REB). Funding for this project was received from the Craig H. Neilsen Foundation. Dr. Michael Fehlings is supported by the Gerald and Tootsie Halbert Chair in Neural Repair and Regeneration and by funding from Phillip and Peggy DeZwirek.

### Segmentation

In this work we analyzed cervical spinal cord MR images from ten healthy volunteers (50% male, mean age = 29.5 years, range = 19 to 47 years). All imaging data was acquired on a 3T GE MR system at the Toronto Western Hospital using an 8-channel neurovascular array coil. Subjects were carefully positioned to limit head movement and were requested to not move. A T_2_-weighted acquisition was obtained to optimize visualization of cervical nerve rootlets emerging from the spinal cord in a segmental fashion. We utilized a FIESTA-C sequence (T_2_-weighted); 512 x 512, NEX 1.0, FOV 200 mm, slice thickness 0.3 mm; resulting in a voxel size of (0.3906 mm x 0.3906 mm x 0.3000 mm). Total scan time was approximately 12 min.

The complete segmentation involves the following steps: creation of a B-spline to approximate the center-line of the spinal cord, segmentation using template matching, an optional smoothing step, and re-centering of the center-line. These are detailed hereafter.

**Fig 1:**
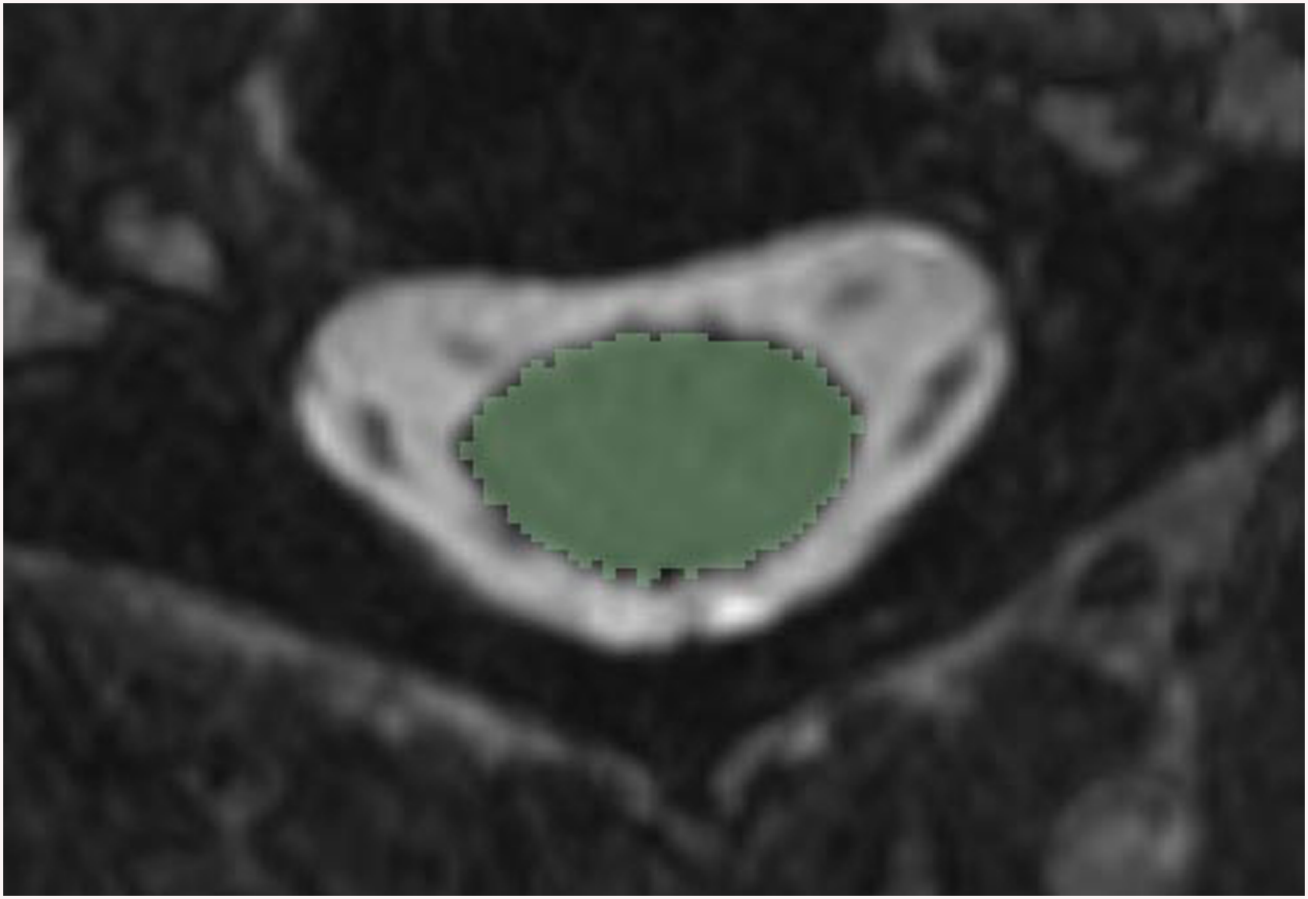
One manually segmented axial slice of a MR image. This image was shown to the raters to instruct them on how to manually segment the spinal cord.

Before proceeding to the automatic segmentation process, ground-truth data was derived from manually segmented spinal cords. Using 3D Slicer (freely available, http://www.slicer.org), a medical image viewer similar to other popular DICOM viewers such as Osirix, “Rater 1” and “Rater 2”, who are both knowledgeable about spinal cord anatomy, were each instructed to manually segment all ten spinal cords, i.e., color in both the grey and white matter of the spinal cord, using a single color, to create a 3D volume mask, from the ponto-medullary junction (PMJ) to any point caudal to the C7 vertebrae. Both raters were instructed to only color in the spinal cord, excluding any nerve roots, CSF, soft tissues, or bony anatomy. An example of the methodology, shown in Fig 1, which illustrates an axial slice of a MR image that has been segmented / colored, was shown to each of the raters to illustrate the technique. The contrast between CSF and the spinal cord was used as a visual guide to distinguish these areas, as well as their expert knowledge in spinal cord anatomy. In areas of low contrast, the raters were required to use their judgment, but were able to view the sequential image slices to make informed judgments. The manually segmented image masks from Rater 1 were chosen to create the ground truth template data for the segmentation of the other images, using a leave-one-out approach. Rater 2 also identified, by selecting either a single point or very small volume on both sides of the spinal cord, the spinal nerve rootlets as they exit the spinal cord for each of the C3–C8 spinal cord segments on all ten subjects. This process creates multiple 3D masks which can be used to determine the precise location of segmentation regions based on nerve rootlet anatomy. These regions are then used to compare cross-sectional area calculations between manual and automatic segmentations, as discussed in later sections of this paper. The identified rootlets are not used for the segmentation process itself.

**Fig 2:**
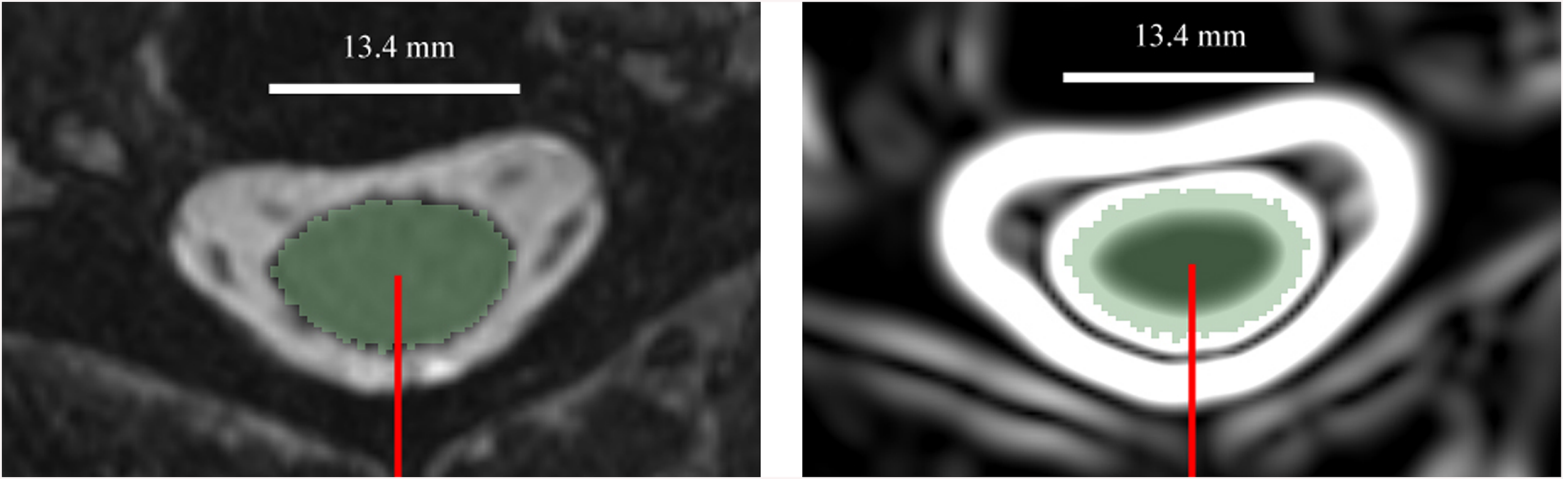
An axial slice of the MR image (left) and the gradient image (right) of the cervical spinal cord. πThe light green shading illustrates the manual segmentation. The red radial line rotating about the spinal cord centerline axis was used for extracting the MRI signal.

To create the ground truth data, one-dimensional template arrays are created from the set of radial lines emanating from the center of the spinal cord of the manually segmented image. The center of the manually segmented spinal cord was calculated by taking the centroid of the segmentation in all axial planes along the length of the cord. Radial lines emanate from the center point in two degree increments in the axial plane. The nearest voxels to the calculated radial line (using 0.1mm increments along the radial line) were recorded. 70 voxels were used to create each radial line. The first image shown in Fig 2, an axial slice of a MR image, shows the segmented spinal cord (shaded light green) and a single radial line emanating from the center of the spinal cord to 70 voxels away from the center of the spinal cord. The second image in Fig 2 shows the magnitude of the gradient of the image (the “gradient image”), for the same axial slice. The gradient image shows areas of contrast, which are more pronounced at the intersection of the CSF and spinal cord.

**Fig 3:**
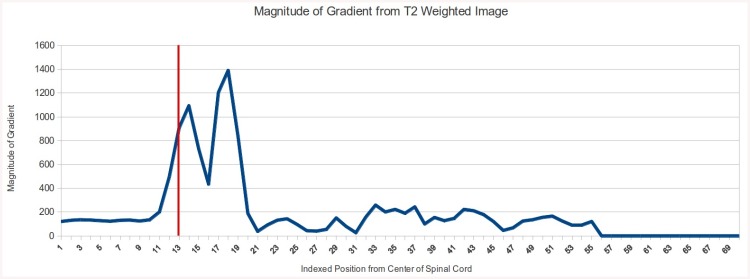
Magnitude of gradient of T_2_ weighted image voxel intensities plotted against the indexed position from the center of the spinal cord. The red line represents the boundary between the spinal cord (voxel intensities plotted to the left of the red line) and the adjacent CSF (voxel intensities plotted to the right of the red line), as indicated by manually segmenting the spinal cord. Values above indexed positions of 56 are zero because this position marked the border of the image for this subject. The double peak represents the consistent area within the CSF where the gradient is diminished. The indexed position is in “voxel”, therefore the template varies with the resolution of the native image. It is however straightforward to express the position in absolute distance units. Also, the magnitude of the gradient depends on the contrast, sequence and manufacturer, but the template matching approach normalizes the signal (because a Pearson’s coefficient is calculated) so that the proposed template can theoretically be applied to any type of data.

Following the radial line from the center of the spinal cord, the location of the spinal cord / CSF boundary is located where the shaded green area stops. This is illustrated graphically in Fig 3, which shows the values for the gradient image along the radial line shown in Fig 2.

The one-dimensional template arrays were created by recording the voxel values of the gradient image along these radial lines and the point at which the edge of the manual segmentation was detected. Approximately 1,000 axial slices were used between the PMJ to caudal of the C7 vertebrae. Within each axial slice, one-dimensional radial arrays were created in two-degree increments (179 radial lines per axial slice). The size of each radial array was set at a fixed distance of 70 voxels (~ 27 mm), while the millimetre distance varied based on the direction of the radial, since all three voxel dimensions are not equal. The set of templates from all of the manually segmented images were used to create the “template database”. The total number of one-dimensional radials recorded was approximately 75,000 from a single manually segmented spinal cord. The number of templates depends on the length of the spinal cord in the image used. The base case analysis utilizes a leave-one-out analysis, using one subject to create the ground truth data to segment the remaining nine subjects. This process is repeated for a total of 10 different sets of templates and 90 total segmentations. Using multiple subjects to create the templates is explored in the results and discussion sections.

The semi-automated segmentation method requires initial user input to approximate the center of the non-segmented spinal cord, between the start and end points of the desired segmentation region, using 3D Slicer. More markings are favorable in areas of high curvature to ensure the approximation stays within the boundary of the spinal cord and as close to center as possible. In this study, we chose to use the PMJ as the starting point in all cases. This was for the purpose of calculating distances to specific nerve root regions from the PMJ, which were also marked for the purpose of this study, but not a requirement of initialization for segmentation. The nerve rootlet regions were used to calculate comparable CSAs across subjects, rather than using vertebral bodies as a surrogate marker. The center-line markings are used to generate a three-dimensional Catmull-Rom spline function (the "spline"), which represents the central axis of the spinal cord, and the center of a generalized cylindrical co-ordinate system. The co-ordinates of the cylindrical system are represented by z (the arc distance along the spinal cord), Ɵ (the rotational angle on a plane orthogonal to the spinal cord axis), and r (the distance from the center of the spinal cord to any radial point at a given z and Ɵ). The spline is a continuous function constructed by joining polynomial functions that are separated by a set of control points. Distance interpolations can be made at any point along the curve to calculate arc lengths between points of interest. The equation for an interpolated spline point is presented below, where ${\left\{P_{i}\right\}}_{i=1}^{N}$ represents the set of N control points (the control points are the 3D spinal cord markings). Twenty control points were used to initialize the spline (when the user initially selects points in 3D Slicer, the paintbrush used is typically larger than a single voxel, so many more than 20 voxels are selected; an equally distributed set of 20 voxels is selected from the larger set of points to create the initial control points). Twenty points was arbitrarily chosen, but is greater than the number of points needed to appropriately mark the curvature of the spine in all ten subjects presented. The number of control points could be adjusted higher or lower if needed. We calculate the position $\vec{p}\left(t\right)$ with the next equation:

$$\vec{p}\left(t\right)={\vec{P}}_{i+1}\cdot h_{00}\left(t^{\prime }\right)+N\left({\vec{P}}_{i+2}-{\vec{P}}_{i}\right)\cdot h_{10}\left(t^{\prime }\right)+{\vec{P}}_{i+2}\cdot h_{01}\left(t^{\prime }\right)+N\left({\vec{P}}_{i+3}-{\vec{P}}_{i+1}\right)\cdot h_{11}\left(t^{\prime }\right);t\in \left[0,\ldots ,1\right)$$

where *i* = *t*⋅(*N*−1), *t′* = *N*⋅*t*−*i* and *h*_*ij*_ are Hermite basis functions. *t* is the control variable, and we assign *t* = 0 to represent the top marking along the spinal cord, the PMJ, and *t* = 1 to represent the last control point (caudal to the C7 vertebrae in this study). In our formulation we assign the control points to be equi-distributed in *t* ∈ [0,…,1]. That is a choice of representation, and the control points themselves do not have to be equi-distributed along the spline (*z* direction). That does not create any problem since *t* is an auxiliary variable and is not used directly. Instead, we use *z* in our formulation, which can be calculated as the arc length of any interval, as explained below.

Notice that *P*_0_,*P*_N+1_, are additional control points that are chosen such that the tangent of the spline in the first/last points would be the direction to the next/previous point, correspondingly. The tangent of the spline can be calculated at any point, *p(t)*, using the interpolated spline point formula shown above, but replacing *h*_*ij*_ with $N\frac{dh_{ij}}{dt^{\prime }}$.

Arc length, *s*, is measured from the spline origin, the PMJ, to any point along the spline using the following equation. *s*(*t*) represents the spline’s length in millimeters, and is referred to as *z* in our generalized cylindrical coordinates system. We can calculate the arc length (*z*) as following:

$$s\left(t\right)=z=\;\overset{t}{\underset{0}{\int }}\sqrt{\vec{p}{\left(t^{\prime }\right)}^{2}}dt^{\prime }$$

To convert a Euclidean point $\vec{x}=\left(X,Y,Z\right)$ to its generalized cylindrical coordinates representation $\vec{c}=\left(z,\theta ,r\right)$, we search for t*, the closest point on $\vec{p}\left(t\right)$, and then calculate the new coordinates

$$\vec{c}=\left(s\left(t^{*}\right),\;arccos\frac{\left(\vec{x}-\vec{p}\left(t^{*}\right)\right)\;{\hat{d}}_{0}}{\left|\vec{x}-\vec{p}\left(t^{*}\right)\right|},\left|\vec{x}-\vec{p}\left(t^{*}\right)\right|\right)$$

Notice that the formulation assumes that the spline’s shape is such that $\vec{x}\;$ lies in the plane perpendicular to $\vec{p}\left(t^{*}\right)$.

Conversion from $\vec{c}$ to Euclidean representation $\vec{x}$ can be done by finding t*, the point where *s*(t*) = *c*_*z*_ and then calculate the Euclidean position.

$$\vec{x}=\vec{p}\left(t^{*}\right)+r*R\left(\theta \right)\left(\frac{\partial \;\vec{p}\left(t^{*}\right)}{\left|\partial \vec{p}\left(t^{*}\right)\right|}\times \left({\hat{d}}_{0}\times \frac{\partial \;\vec{p}\left(t^{*}\right)}{\left|\partial \vec{p}\left(t^{*}\right)\right|}\right)\right)$$

where *R*(*θ*) is a 2x2 rotation matrix. Notice that we assume that $\vec{p}$ is never parallel to ${\hat{d}}_{0}$.

Using a spline function to represent the spinal cord allows us to utilize the generalized cylindrical coordinate system as explained above.

The template database was used to segment new images, using a leave-one-out analysis as previously described. The semi-automatic method begins with an initial center-line approximation made on the non-segmented image, as outlined above, and the calculation of the image gradient. 17,900 “test” arrays were taken from the image, consisting of 179 radials from each of 100 axial slices, evenly spaced down the spinal cord from the first user marking, the PMJ, to below the C7 vertebrae. Normalized cross-correlation was used to compare the similarity in the shape of the “template” database arrays and the “test” arrays. For arrays with a certain degree of similarity in shape, we infer that the location of their spinal cord / CSF boundary is located at approximately the same distance from the center of the spinal cord in both the “test” and “template” arrays.

Starting with the “test” arrays located at an arc distance of 0 mm from the starting point and working caudally, each radial “test” array was compared against all of the “template arrays” in the template database according to the following formulas:

$$Normalized\;Cross\;Correlation=\;\frac{1}{n}\Sigma \frac{\left(f\left(x\right)-\bar{f}\right)\left(t\left(s\right)-\bar{t}\right)}{\sigma_{f}\sigma_{t}}.$$

$$\sigma_{f}=\;\sqrt{\frac{1}{n}\Sigma {\left(f\left(x\right)-\bar{f}\right)}^{2}}$$

and

$$\sigma_{t}=\;\sqrt{\frac{1}{n}\Sigma {\left(t\left(x\right)-\bar{t}\right)}^{2}}.$$

Where *f* and *t* are the “template” and “test” arrays.

**Fig 4:**
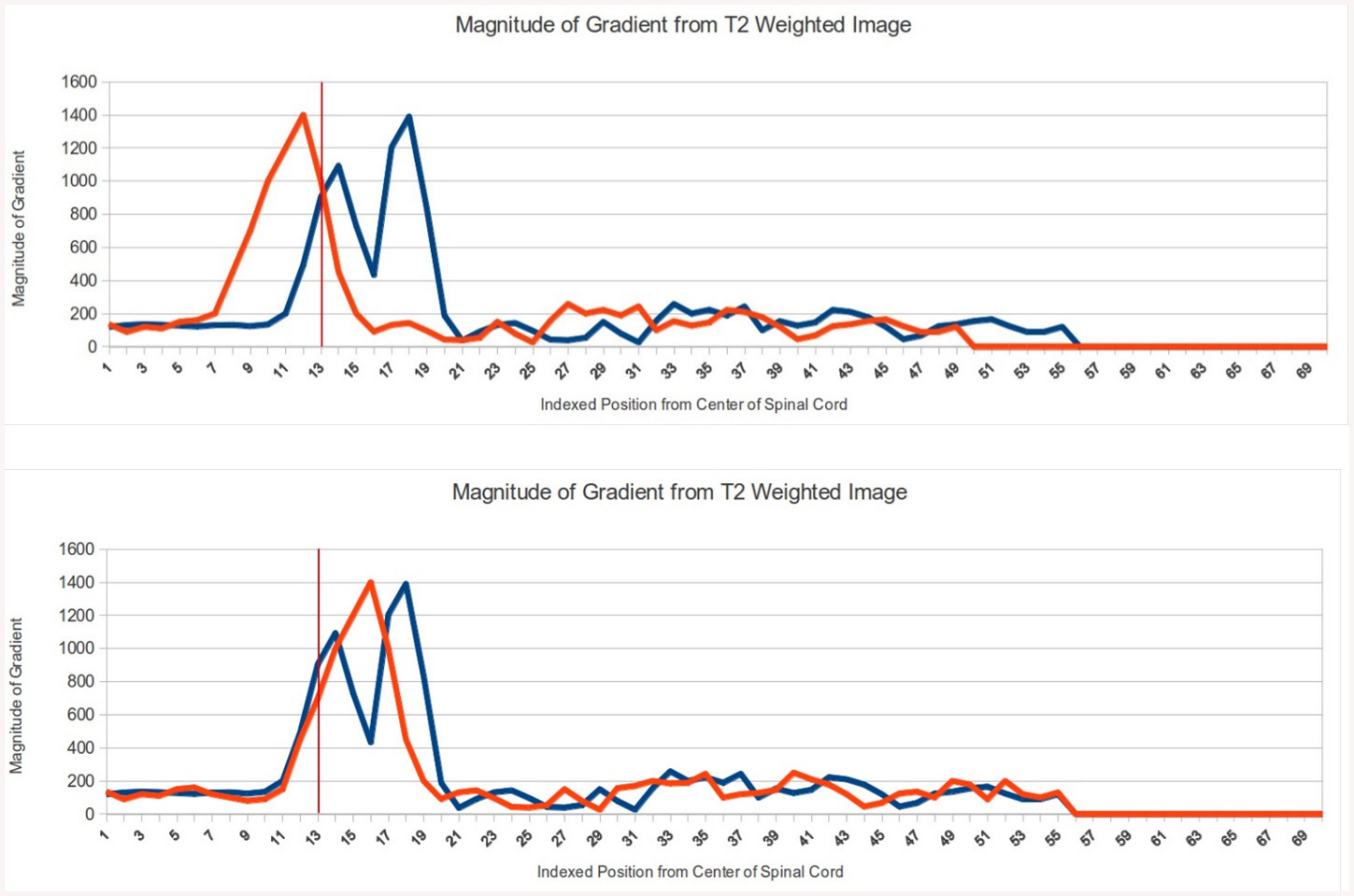
An example of normalized cross correlation for two different “test” arrays with the same template array. The first graph above is a test array (red) with a cross-correlation value of 30% to the template array (blue). The second graph is a test array (red) with a cross-correlation value of 74% to the template array (blue). The matching algorithm starts with a threshold of 100% and identifies matching templates. The algorithm aims to find 50 matching templates at the highest threshold, an arbitrary number of matches that was found to produce suitable results. If 50 were not found at a given threshold level, the threshold was decremented by 200 basis points (e.g., 100% threshold drops to 98%). The 50 matching templates were then used to infer the distance to the spinal cord edge of the test image by using the average of the distances from each of the matching templates.

The output of each template comparison is the cross-correlation value, or the percentage similarity between the template and test array. Examples of two matches are shown in Fig 4. The first has a 30% cross-correlation and the second has a 74% cross-correlation.

**Fig 5:**
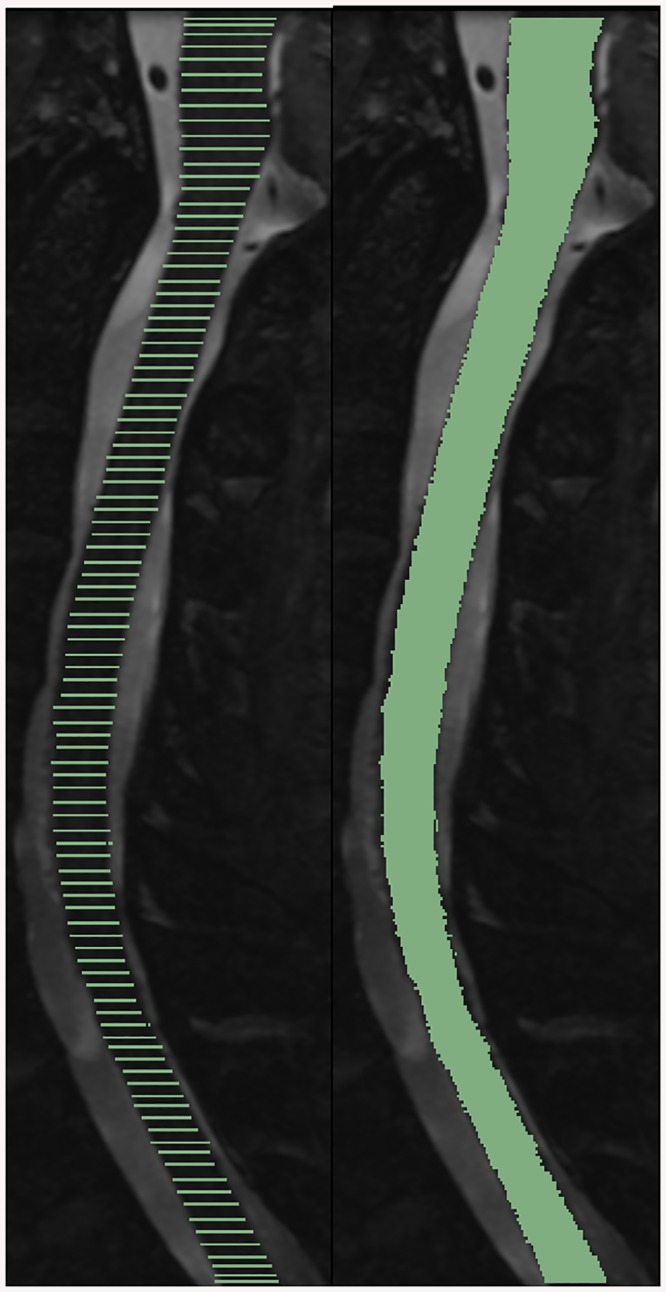
A mid-sagittal semi-automatically segmented image at 100 points along the Z-axis shown before (left) and after interpolation (right).

The semi-automatic spinal cord / CSF boundary finding algorithm was applied to only 100 axial planes to reduce computation time. To locate the spinal cord / CSF boundary of the remainder of the spinal cord, the edge information was linearly interpolated. The output is a segmented spinal cord image between the regions that were manually initialized. The segmented region can be used to make volume calculations and measure distances, as described below. A 2D sagittal slice of a partially segmented spine (after the initial template matching) and a fully segmented spinal cord (after interpolation) is shown in Fig 5.

To improve the arc distance measurements along the longitudinal axis of the spinal cord, a set of refined control points was calculated. This was done by finding the centroid of each segmented axial plane (roughly 200–300 axial slices per image, depending on the length of the spinal cord), then setting these centroids as control points of the spline. These points are more accurately centered than the original approximation which was made manually in Part A. The new number of control points was arbitrarily set at 500, which ensures that all of the axial planes have at least one control point assigned.

When areas of low CSF volume are encountered, the template matching algorithm may not find a match which meets the minimum threshold, or may find many unsuitable matches if using a lower threshold. This pattern is observed when the CSF space is either diminished or obliterated as is the case in some normal individuals with a congenitally narrow spinal canal or in cases of pathology such as a disc herniation. To attempt to remove these outliers, a series of one dimensional median smoothing filters can be applied in a cascade, if warranted. The filters are applied to the one-dimensional arrays of newly located edge distances, in two different planes: 1) in the axial plane, and 2) along the spinal cord, in the z-direction, holding Ɵ constant. Smoothing is not applied in the base case analysis, but the impact of applying the smoothing filter is assessed in the discussion section.

In total, the segmentation process takes approximately 1.5 to 2.0 minutes, depending on the subject. The segmentations were performed on an Intel Core i7 processor with no thread optimization or GPU acceleration. All algorithms were written using a combination of Python and C++, with use of the ITK imaging software package (www.itk.org), Numpy (www.numpy.org), and Scipy (www.scipy.org).

### Validation

To validate the semi-automatic segmentations, ten subjects were manually segmented by Rater 1 and Rater 2, using 3D Slicer, and compared against the semi-automatic segmentations of the same subjects. Importantly, the manual segmentations from Rater 1 only were used to generate the templates. We also show two cases where two subjects were used in combination to create the templates, instead of one.

The segmentation quality was assessed by:

1. The total 3D segmentation overlap, as measured by the Dice co-efficient (DC):
  $$Dice\left(A,B\right)=2\left|\frac{A\cap B}{\left|A\right|+\left|B\right|}\right|$$
2. The mean distance (MD), representing the average distance between the two segmentations’ spinal cord center line on each axial slice. This measures the ability to accurately determine spinal cord arc distances.
  $$MD=\frac{\sum_{0}^{n}\left(p_{2}-\;p_{1}\right)}{n-1},$$
  where (p2—p1) is the Euclidian distance between the center of the splines on each axial slice and n is the number of axial slices in the region of interest.
3. The Hausdorff distance (HD), the maximum distance between two segmentation surfaces or 3D volumes[14]. The HD captures large outliers segmentation points produced by low CSF volume or spinal cord deformities. *HD* = *max*(*D*(*S*_1_,*S*_2_),*D*(*S*_2_,*S*_1_)), where *D*(*S*_1_,*S*_2_) = max*d*(*p*,*S*_2_) and *d*(*p*,*S*_2_) = min *dist*(*p*,*p* ^1^) is the distance between a point and a surface S_2_
4. The difference in cross sectional area (CSA) of the spinal cord between the manual and the automatic segmentations. This will capture the ability of the segmentation to measure small differences in the segmentation at the outer edge of the cord, where small differences in cord radius present as large differences in area.
  $$CSA=\frac{\Sigma segmented\;voxels\;in\;a\;region}{Arc\;length\;of\;region}\;\times Voxel\;Size,$$
  and
  AbsoluteDifferenceInCSA=|CSA2−CSA1|.

The C3–C8 nerve root segments were manually marked by Rater 2 in 3D Slicer. These points served as boundary markings. The segmented volume and arc length distance between the orthogonal planes at the most rostral and caudal points of these areas were used to calculate average CSA. Note that the same spline, calculated from the manual segmentation, was used to determine the location of the desired target areas in both volumes being compared. Inter-rater validation was performed on n = 10 subjects. Intra-rater reliability was not assessed.

Several modifications were made to the base case, including smoothing the segmentations and adjusting the size of the ground truth data set. Smoothing applies a median smoothing filter with a fixed kernel size (ranging from five to nine voxels) to the segmented axial arrays in order to eliminate outliers. This was accomplished both in the axial plane and along the longitudinal axis of the spinal cord, the z axis. The impact of doubling the size of the ground truth data set was also analyzed. Two variations of modified ground truth data were made, based on: 1) using the two sets of ground truth data that yielded the overall lowest Dice co-efficient values, and 2) using the two sets of ground truth data that yielded the overall highest Dice co-efficient values. These results are shown as modifications to the base case.

## Results

**Fig 6:**
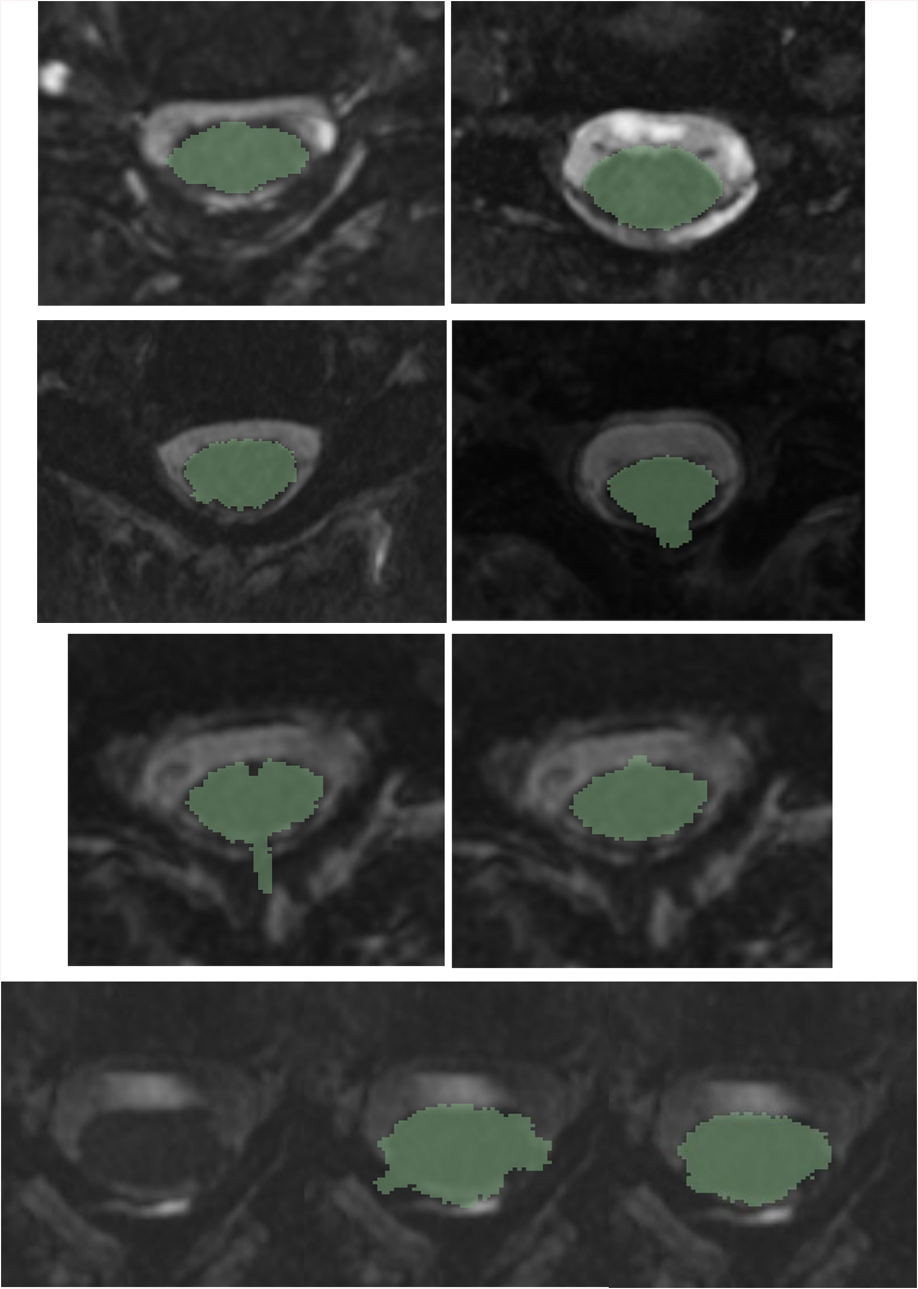
Examples of difficult segmentations due to reduced CSF, artifacts, and spinal rootlets. In the first four examples, the segmentation performed well. In the next two examples (third and fourth rows), applying a smoothing filter improved the segmentation result due to reduced CSF volume and image artifacts.

Examples of axial plane segmentations are shown below in Fig 6. In the first four examples, the algorithm was able to perform a suitable segmentation based on the ground-truth data available in a difficult image. These images are difficult due to the reduced CSF, artifacts in the CSF, and the spinal rootlets exiting the cord (most notably in the top right image). In the fifth image (third row, left), the segmentation did not work as well due to a complete obliteration of the CSF, as well as the tissue outside of the spinal cord producing an undesirable gradient. In these cases, using the smoothing process may be beneficial. The smoothed segmentation for this subject is shown in the sixth image (third row, right). Another example of this is shown in images seven through nine (bottom row). The original image has a complete obliteration of the CSF in several locations (bottom left). The un-smoothed segmentation is shown in the middle image. Applying the smoothing filter (bottom right) improved the results significantly. The impact of applying the smoothing process to all of the subjects is illustrated in Table 1 below.

**Table 1:** Spinal Cord Segmentation Validation Results.

	DC (% similarity)	MD (mm)	HD (mm)
**No Smoothing—Base Case**			
**R1 vs R2 (n = 10)**			
Mean +/- Standard Deviation	0.92 +/- 0.02	0.29 +/- 0.07	2.09 +/- 0.38
Min/Max	0.88 / 0.94	0.17 / 0.41	1.75 / 2.94
**R1 vs SA (n = 10)**			
Mean +/- Standard Deviation	0.91 +/- 0.03	0.28 +/- 0.07	1.91 +/- 0.54
Min/Max	0.84 / 0.96	0.15 / 0.52	1.19 / 3.37
**R2 vs SA (n = 10)**			
Mean +/- Standard Deviation	0.91 +/- 0.02	0.32 +/- 0.08	1.82 +/- 0.33
Min/Max	0.83 / 0.95	0.17 / 0.55	1.25 / 2.51
**Adjustments to Base Case**			
**R1 vs SA (n = 10)–With Smoothing**			
Mean +/- Standard Deviation	0.91 +/- 0.03	0.27 +/- 0.07	1.85 +/- 0.60
Min/Max	0.83 / 0.96	0.14 / 0.51	1.06 / 3.94
**R1 vs SA (n = 10) Two Subject Ground Truth Data (Worst)–No Smoothing**			
Mean +/- Standard Deviation	0.89 +/- 0.03	0.30 +/- 0.06	2.04 +/- 0.65
Min/Max	0.85 / 0.94	0.22 / 0.41	1.19 / 3.37
**R2 vs SA (n = 10) Two Subject Ground Truth Data (Best)–No Smoothing**			
Mean +/- Standard Deviation	0.92 +/- 0.01	0.25 +/- 0.05	1.84 +/- 0.66
Min/Max	0.90 / 0.93	0.16 / 0.31	1.26 / 3.17

Top: Validation of spinal cord segmentation. Comparisons are made between Rater 1 (R1), Rater 2 (R2), and the semi-automatic (SA) methods. The 3D Dice coefficient (DC) measures the amount of overlap between two 3D volumes; mean distance (MD) measures the average distance between the spinal cord center-line in all axial planes of the image; Hausdorff Distance (HD) measures the maximum distance between the two 3D surfaces.

Bottom: Adjustments made to the base case segmentation. First, the impact of using a cascade of smoothing filters with different kernel sizes on the segmentation metrics is shown. Second, the impact of using two subjects to create the ground truth data is shown, using both the set of the worst two performing sets of data and the best two performing sets of data.

The results of the base case segmentation (no smoothing, one set of ground truth data) are shown in the top part of Table 1. Comparisons are made between Rater 1 (R1), Rater 2 (R2) and the semi-automatic algorithm. Adjustments to the base case are shown in the bottom part of Table 1. The DC values shown represent the average DC across all subjects (n = 10). The minimum and maximum values represent the DC of a single subject, but not necessarily the same subject. The MD and HD are calculated at multiple points for each subject and averaged, resulting in a single value for each subject. The minimum and maximum values are recorded for each subject. The results shown below for MD and HD are the average of the individual averages (n = 10). The minimum and maximum represent the absolute minimum and maximum across all subjects (n = 10), and may not correspond to the same subject.

The first part of Table 2 shows the average CSA for various spinal cord segments. Note that these are not the areas of the spinal cord adjacent to the corresponding vertebrae, rather, they are the spinal cord segments demarcated by the spinal rootlets branching from the cord. The spinal rootlets were accurately marked and validated by two raters with anatomical spinal cord knowledge. This is intended to show the average size of each spinal cord region and to demonstrate the variability in spinal cord cross-sectional area across a small group of subjects.

**Table 2:** Analysis of Spinal Cord Cross-Sectional Area (CSA).

Mean CSA +/- Standard Deviation (mm^2^)						
	C3	C4	C5	C6	C7	C8
R1 (n = 10)	75.73 +/- 9.19	80.14 +/- 12.93	84.95 +/- 14.06	88.64 +/- 15.09	82.68 +/- 14.24	65.62 +/- 8.46
R2 (n = 10)	77.90 +/- 9.89	78.90 +/- 12.44	84.02 +/- 14.19	85.39 +/- 15.80	77.97 +/- 16.57	65.00 +/- 11.89
Semi-Automatic	75.23 +/- 6.85	76.74 +/- 9.35	82.22 +/- 11.37	83.14 +/- 11.93	79.14 +/- 12.36	68.80 +/- 10.04
**Average Absolute Difference in CSA Between Methods, by Spinal Cord Segment (mm** ^**2**^ **)**						
	C3	C4	C5	C6	C7	C8
R1 vs R2(n = 10)	7.74	7.41	8.49	9.86	9.31	7.94
R1 vs SA(n = 10)	7.22	8.59	7.32	10.67	10.63	7.88
R2 vs SA (n = 10)	4.33	5.98	5.43	8.25	8.80	6.23

Top: Cross-sectional areas (CSA) were calculated for the same spinal cord segments (as determined by Rater 2 marking the nerve rootlets coming off of the spinal cord in each segment) and compared across subjects.

Bottom: The absolute difference in CSA between two segmentations, averaged across all of the subjects within that group.

The second part of Table 2 shows the absolute difference in CSA between two segmentations, averaged across all of the different subjects. For example, to calculate the difference in C3 between R1 and SA, the C3 area for subject 1 from each of the nine segmentations was averaged. Next, the absolute difference between that average and the CSA determined by R1 was calculated, for each of the 10 subjects. These differences were then averaged and are shown in the table.

**Fig 7:**
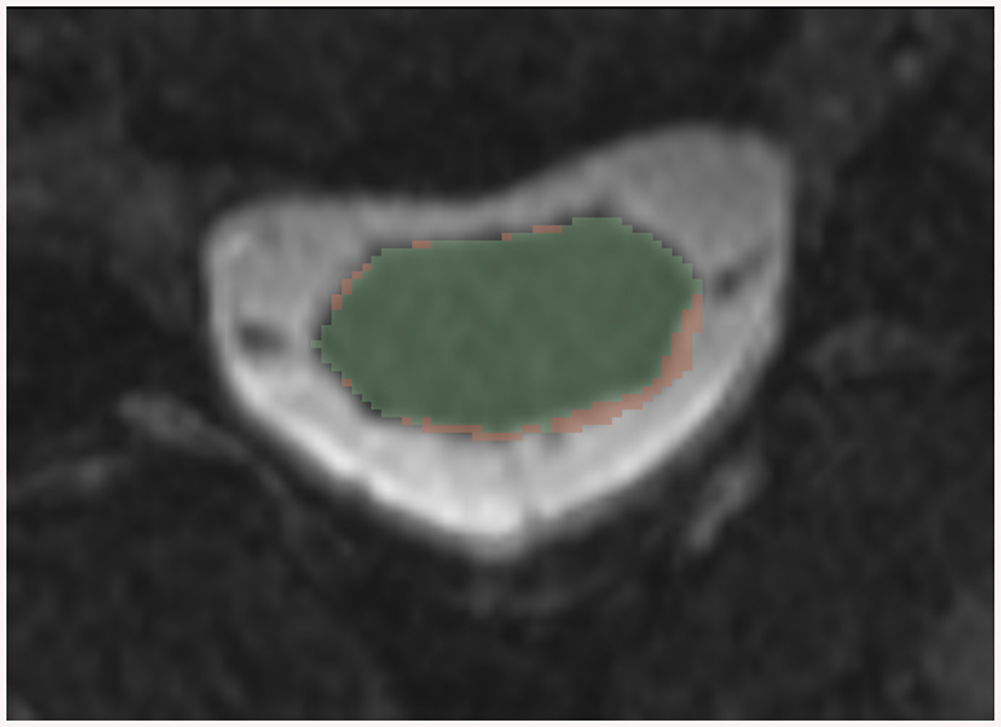
Example of the difference between two raters segmentation of a smoothed T2-weighted MR image. The green region represents the area segmented by Rater 1 and the red region is Rater 2 less Rater 1. Although the segmentations are very similar, and both could subjectively be considered accurate, there is an 8 mm^2^, or 9%, difference in CSA.

In Fig 7, we illustrate the difference in segmentation between two manual segmentations. The area in green represents the area marked by Rater 1 and the area in red represents Rater 2 minus Rater 1. Although this difference looks small, it represents an 8 mm^2^ CSA difference, or approximately 10% of the CSA.

Lastly, Table 3 shows a comparison of these results to prior studies, where data is available.

**Table 3:** Comparison of Current Segmentation Method to Prior Studies that Calculate Comparable Metrics.

	Voxel Dimensions (mm)	Inter-Rater		Automated vs. Rater	
		Dice (% similarity)	Hausdorff (mm)	Dice (% similarity)	Hausdorff (mm)
Current Method (#)					
T2 (n = 10)*	0.39 x 0.39 x 0.30	0.92 +/- 0.02	2.09 +/- 0.38	0.91 +/- 0.02	1.82 +/- 0.33
Chen et al.,2013 [3]					
T1 (n = 7)	1.0 x 1.0 x 1.0	0.89 +/- 0.02		0.82 +/- 0.18	
MT (n = 4)	0.6 x 0.6 x 2.25	0.93 +/- 0.01			
MT (n = 20)^	0.6 x 0.6 x 2.25			0.92 +/- 0.01	
De Leener et al., 2014 [2]					
T1 (n = 5)	1.0 x 1.0 x 1.0	0.86 +/- 0.05	2.18 +/- 0.38	0.88 +/- 0.02	2.21 +/- 0.33
T1 (n = 15)	1.0 x 1.0 x 1.0			0.90 +/- 0.03	2.44 +/- 0.81
T1 (n = 5, Horsfield) (#)	1.0 x 1.0 x 1.0			0.88 +/- 0.02	1.87 +/- 0.51
T1 (n = 15, Horsfield) (#)	1.0 x 1.0 x 1.0			0.89 +/- 0.03	1.85 +/- 0.23
T2 (n = 5)	1.0 x 1.0 x 1.0	0.91 +/- 0.02	1.88 +/- 0.45	0.88 +/- 0.03	1.86 +/- 0.42
T2 (n = 15)	1.0 x 1.0 x 1.0			0.90 +/- 0.03	2.01 +/- 0.67
T2 (n = 5, Horsfield) (#)	1.0 x 1.0 x 1.0			0.86 +/- 0.03	2.25 +/- 0.76
T2 (n = 15, Horsfield) (#)	1.0 x 1.0 x 1.0			0.88 +/- 0.03	1.92 +/- 0.24
Koh et al., 2010 [11] (#)					
T2 (n = 55)*	4.5 thickness			0.70 +/- 0.05	
Koh et al., 2011 [12]					
T2 (n = 60)*	4.5 thickness	0.90 +/- 0.02		0.71 +/- 0.06	

*–Represents the best outcome based on comparison between two manual raters

^^^–Represents the best outcome from two different methods

^#^–Denotes methods where user initialization is required

Note: In the above table, the Dice coefficient depends on the spatial resolution, which in our case is favourable, however the Hausdorff distance does not, and therefore results suggest high performance of the proposed method.

McIntosh and Hamarneh also calculated an automatic versus manual maximum inter-surface distance of 1.095 mm, on average, but it is not clear if this method is comparable to the Hausdorff distance.

## Discussion

This study presents a novel method of semi-automatic segmentation of the human spinal cord using MRI data. The method requires minimal user-input and takes under 2 minutes of computation time to segment the cervical spinal cord on an image with a 0.3906 x 0.3906 x 0.3000 mm voxel size. The algorithm performs well compared to other segmentation methods, including having the lowest average Hausdorff distance, an average Dice coefficient only 100 basis points less than comparing to a second manual rater, or the next best algorithm. The Hausdorff distance illustrates that there is less distance between surfaces when comparing semi-automatic to manual segmentations: 1.82 mm / 1.91 mm in the base case, and 2.09 mm, respectively. Lastly, the mean distance between spinal cord center lines is less than two voxels in all cases, illustrating that the semi-automatic method works as well as manual segmentation when finding the center line of the spinal cord.

The results at the bottom of Table 2 illustrate how the difference between the CSA of SA and manual segmentations are rarely greater than the differences in CSA between two manual raters. This highlights the accuracy of the semi-automatic method for new segmentations. In the cases where the manual raters had less absolute error, the difference was not large.

Novel to this analysis are the nerve rootlet markings we used to accurately identify spinal cord segments. We believe this more accurately represents specific anatomical regions within the spinal cord. For example, the C5 spinal cord segment is known to contain lower motor neurons that sub serve the function of the deltoid muscle. In performing the analysis in this way, we aim to eliminate any potential confounding errors associated with measuring regions of the spinal cord that contain different cellular constituents. We believe this improves the relevancy of the CSA results compared to prior studies which measure CSA.

Calculating CSA is difficult for two reasons: 1) the resolution of the image can be very important in determining the accuracy of the CSA calculation (small segmentation errors in low resolution images exaggerate CSA differences); and 2) manual segmentation of the spinal cord is subjective. It is difficult to precisely demarcate the dividing line between the spinal cord and CSF, particular when segmenting a large area of the cord, a very time consuming process. An example of this issue is shown in Fig 7. This is the source of the inter-rater segmentation differences. Lastly, small segmentation differences can exaggerate CSA differences because the changes occur at the margin of the segmentation, where the CSA is roughly equal to the square of the incremental radius (assuming a circular spinal cord to simplify). For example, a one voxel difference at the margin of a spinal cord with a 5 mm radius can result in an area difference of 16 mm^2^, or 17%. This is one reason for variation in our results.

In the set of ten subjects segmented by the semi-automatic method, several had reduced CSF volumes, and artifacts which appeared as spinal rootlets, but none had a completely diminished CSF space. For this reason, the segmentation worked well without applying the smoothing process. We believe reduced CSF and artifacts are the main source of segmentation error in this study. Using the smoothing method described above works well in the images which have significant areas of error due to reduced CSF, or very dark spinal rootlets, but as a group, smoothing did not improve the segmentation results significantly. It improved the average Hausdorff distance from 1.91 mm to 1.85 mm and the mean distance between spinal cord center lines from 0.28 mm to 0.27 mm, but had no impact on the Dice coefficient.

The choice of subject to create the ground-truth data did impact the results. When comparing Rater 1 to the semi-automatic segmentation, the range of Dice coefficients was 0.84 to 0.96, the range of Hausdorff distances was 1.19 to 3.37, and the range of mean distances was 0.15 to 0.52. Using a larger set of the “best” ground truth data, these ranges were 0.90 to 0.93, 1.26 to 3.17, and 0.16 to 0.31, respectively. All of the ranges improved. The results shown in Table 1 illustrate that you can achieve more or equally accurate results using only one segmentation instead of the average of 90 segmentations.

The segmentation process relies on user initialization of the spinal cord center line. The reproducibility of the analysis was assessed by comparing the segmentations produced by two different initializations. All 10 subjects were initialized by both raters, then processed by the segmentation algorithm using the ground truth data of one subject, which was arbitrarily chosen. The automatic segmentations were then compared using the same metrics as the segmentations presented in the results section. The average and standard deviations for DC, MD, and HD were 0.96 +/- 0.01, 0.13 +/- 0.05 mm, and 1.61 +/- 0.64 mm, respectively. The average values were improved in all cases compared to the manual segmentation metrics. This demonstrates that variability in the initialization does not significantly impact the results of the segmentation.

Image voxel size and changing other acquisition parameters may have an impact on the segmentation. This study used small voxel size / high resolution images, which is a requirement for visualizing the spinal nerve rootlets. This algorithm has not been tested on lower resolution images. It is feasible that the ground truth data created in this project could be scaled down to lower resolution images and used to segment those images, but the accuracy has not been tested. Changing acquisition parameters may impact the tissue signals and thus impact the segmentation results. All images in this study were acquired using the same parameters. It is feasible, and not overly time consuming, to create a new set of ground truth data that could easily be used with the same algorithm and new data, should different acquisition parameters be needed which are incompatible with existing images.

Subject pathology may effect the results. For instance, subjects who have a lesion in their white matter may have MR images which produce gradients within the spinal cord. To account for this, a robust ground truth data set would need to be created when implementing this algorithm clinically. This is one of the benefits of this method. It is adaptable for different clinical context and can evolve for different patient populations by changing, or adding to, the set of ground truth data. Alternatively, using the smoothing method described in this analysis would also eliminate the effects of this pathology.

Advantages of this method include its fast processing time, and its ability to work well with high spinal cord curvature and low CSF volume. Although it was not studied, it is also likely this method would work well with T1-weighted MR images, given their similar gradient profiles. It may also work well on other structures that have high contrast relative to surrounding structures, such as contrast images of blood vessels. Disadvantages of this method include the need to initialize the segmentation manually and the need to create a template database from a fully segmented manual spinal cord before subsequent segmentations can be performed.

## Conclusion

Here we present a novel approach to segmenting the human spinal cord from high resolution T2-weighted magnetic resonance images. A one-dimensional template matching algorithm is applied to segment new images. The center-line of the segmented image is corrected based on the new segmentation. Accurate distance and volume/area calculations can be made on the segmented image. This method provides very accurate results when compared to manual segmentation. It utilizes ground truth data to inform the segmentation, as opposed to strictly pure image based methods. It performs well in areas of low CSF volume and in areas with distortions or artifacts within the CSF. This method offers researches a novel way to process and interpret spinal cord imaging data for use in clinical studies that aim to compare metrics across subjects or across groups of subjects.
